# Personality traits and the degree of work addiction among Polish women: the mediating role of depressiveness

**DOI:** 10.1192/j.eurpsy.2025.942

**Published:** 2025-08-26

**Authors:** A. M. Cybulska, D. Schneider-Matyka, E. Grochans, K. Rachubińska

**Affiliations:** 1 Department of nursing, Pomeranian Medical University in Szczecin, Szczecin, Poland

## Abstract

**Introduction:**

Workaholism is an addiction, however the obsessive-compulsive components alone may prove insufficient in determining its nature.

**Objectives:**

The aim of the following study was to determine the mediating role of depressiveness in the relationships between workaholism and personality traits according to the five-factor model among Polish women.

**Methods:**

The research study was carried out among 556 women residing in the West Pomeranian Voivodeship in Poland. The research was based on a survey performed using a questionnaire technique. The following research instruments adapted to Polish conditions were employed to assess the incidence of work addiction among female adults: The NEO Five-Factor Inventory (NEO-FFI), The Work Addiction Risk Test (WART) Questionnaire, and The Beck Depression Inventory–BDI I-II.

**Results:**

A positive correlation between the intensity of neuroticism and the work addiction risk was revealed (β = 0.204, *p* < 0.001). A partial mediation (35%) with the severity of depression symptoms as a mediating factor was observed (β = 0.110, *p* < 0.001). Respondents characterized by high neuroticism showed a greater severity of the symptoms of depression (β = 0.482, *p* < 0.001), which is a factor increasing the work addiction risk (β = 0.228, *p* < 0.001). Respondents characterized by a high level of extraversion displayed lower severity of the symptoms of depression (β = –0.274, *p* < 0.001). A negative correlation between the intensity of agreeableness and the work addiction risk was revealed (β = –0.147, *p* < 0.001). A partial mediation (27.8%) was observed. A positive correlation between the intensity of conscientiousness and the work addiction risk was revealed (β = 0.082, *p* = 0.047). Respondents characterised by a high level of conscientiousness showed a lower severity of depression symptoms (β = –0.211, *p* < 0.001).

**Table 1.** Indirect and total effects: Mediation model 1 - Neuroticism

**Table:** 

	95% CI*						
Type	Effect	b	Lower	Upper	β**	z	*p*-value
Indirect	N ⇒ BDI ⇒ WART	0.149	0.092	0.213	0.110	4.800	<0.001
Component	N ⇒ BDI	0.241	0.205	0.275	0.482	13.270	<0.001
	BDI ⇒ WART	0.618	0.398	0.851	0.228	5.230	<0.001
Direct	N ⇒ WART	0.277	0.157	0.403	0.204	4.540	<0.001
Total	N ⇒ WART	0.426	0.319	0.534	0.314	7.790	<0.001

NEU—neuroticims, WART—Work Addiction Risk Test, N – Neuroticism, BDI—Beck Depression Inventory–BDI I-II, b—unstandardized regression coefficient, β—standardized regression coefficient, *p*—significance level; * Confidence interval (CI) computed with method: bootstrap percentiles; ** Beta (β) is completely standardized effect size.

**Conclusions:**

Depressiveness plays the role of a mediator between neuroticism, extraversion, agreeableness as well as conscientiousness, and work addiction. Depressiveness is a factor which increases the risk of work addiction.

**Disclosure of Interest:**

None Declared

